# Characterization, mechanical, and antibacterial properties of nanofibers derived from olive leaf, fumitory, and terebinth extracts

**DOI:** 10.3906/kim-2003-45

**Published:** 2020-08-18

**Authors:** Nilşen SÜNTER EROĞLU, Suat CANOĞLU, Metin YÜKSEK

**Affiliations:** 1 Institute of Pure and Applied Science, Marmara University, İstanbul Turkey; 2 Department of Textile Engineering, Faculty of Technology, Marmara University, İstanbul Turkey

**Keywords:** Electrospinning, nanofiber, herbal extracts, antibacterial

## Abstract

In this study, nanofiber structures were obtained with convenient polymers (PVA [polyvinyl alcohol] and PCL [poly ^o^-caprolactone]) derived from the herbal extracts of olive leaves, fumitory, and terebinth plants. Optimum nanofiber structures were identified by measuring viscosity and conductivity values and performing morphological analysis, characterization, and mechanical tests of the prepared solutions. The potential use for wound healing at the most efficient level was determined as a result of antibacterial analysis of the structures obtained. APT (PVA/terebinth) and BFO (PCL/fumitory) nanofibers had the thinnest diameter range and the highest strength values. In terms of the determination of antibacterial effects, nanofiber structures of all 3 plant species proved to be effective against bacteria. The greatest effect was observed against *Escherichia coli* in the nanofiber structure containing olive leaves, with a zone diameter of 32 mm. In addition, APT and BFO nanofibers had the highest values of thinness and strength. In these 2 samples, using BFO against *Staphylococcus aureus* and APT against *Candida albicans* increased their areas of activity. In the literature review, no study was available about obtaining nanofibers, especially from fumitory and terebinth plants. This study aimed to increase knowledge on obtaining nanofiber structures, including various polymers derived from olive leaves, fumitory, and terebinth plants.

## 1. Introduction

According to the World Health Organization (WHO), around 20,000 plants are used in healthcare, and 25% of the ingredients used in pharmaceuticals are plants. These numbers are expected to increase in the future [1,2]. The fact that different plants grow in different locations and that each plant may have several different effects, as well as the potential to obtain biocompatible, sustainable, and environmentally friendly products, has encouraged researchers to work in this field.

There are many plants that stand out with their positive effects on human health. One of them is the olive ( *Olea europaea* ) tree, long known as a symbol of the Mediterranean region. Many in vitro and a limited number of in vivo studies have been carried out on the antioxidant, antibacterial, and antimicrobial effects of olive leaves due to the phenolic components they contain. Low molecular weight polyphenols (up to 60–90 mg/g dry leaf weight) in olive leaves, especially oleuropein, have many positive effects. These beneficial properties include antioxidant, antimicrobial, antiproliferative, antiviral (including HIV), antiinflammatory (such as inhibition of 5-lipoxygenase enzyme), antiaging, anticancer, hypoglycemic, and hypocholesterolemic effects [3–8]. In addition, oleuropein has a structure that is effective against both carcinogenic substances and Alzheimer’s disease [9].

The second plant used in the research is the terebinth ( *Pistacia terebinthus* ) of the Anacardiaceae (gum tree and pistachio) family. Different parts of plants of this species have been used for their aphrodisiac, antiseptic, antihypertensive, and gastrointestinal effects and for dental treatment, and for the treatment of wounds and burns, rheumatism, and liver, urinary, and respiratory tract disorders [10,11]. Many researchers have observed that terebinth fruit has antioxidant, antimicrobial, antiviral, anticholinesterase, antiinflammatory, antinociceptive, antidiabetic, antitumor, antihyperlipidemic, antiatherosclerotic, and hepatoprotective effects [10,12–16].

The last type of plant used in this research is the fumitory ( *Fumaria officinalis* ). Also known as the ‘fox’s coriander’ in Turkey, it can grow in all geographic regions [17]. Extracts obtained from the fumitory are used in the treatment of liver dysfunctions, gastrointestinal diseases, eczema and dermatitis, digestive disorders, colic, fever, and migraine [18–20]. In addition, it is known that the fumitory has therapeutic effects thanks to its antibacterial properties.

Nanofibers are produced by several techniques such as drawing, template synthesis, phase separation, self- assembly, and electrospinning. Electrospinning has many advantages, such as having potential for industrial processing, repeatability, convenience for processing, control of fiber dimensions, scalability of the production process, being cost-effective, and allowing the production of long and continuous nanofibers [21]. When all of these properties are evaluated, it is evident that it would be appropriate to produce a nanofiber for wound healing by electrospinning. In recent years, the interest in polymer materials obtained by the electrospinning method has increased considerably. Many researchers [9,22–23] have produced a polymer-containing structure loaded with different plant extracts and have provided ideas for their application in the areas of tissue engineering applications, wound dressing materials, and drug delivery systems.

PVA (polyvinyl alcohol) is used for purposes such as textile sizing, adhesives, protective colloids for emulsion polymerization, paper sizing, medical and food applications, soil stabilizer, wood preservatives, etc. [24]. PVA is preferred because of its semicrystalline, water-soluble, chemical durability, biocompatibility, and good mechanical and film-forming properties as an easily modified secondary hydroxyl group [25]. While fully hydrolyzed grade types of PVA are soluble in hot water, partially hydrolyzed grade types of PVA are soluble in water at room temperature [24]. PVA is generally composed of hydrolysis of poly (vinyl acrylate) (PVAcr) monomer [25]. The hydrolysis process is a partial replacement of the ester group in vinyl acetate with the hydroxyl group and requires aqueous sodium hydroxide [26]. PCL (poly [^o^-caprolactone]) has been successfully incorporated in medical applications thanks to its low melting temperature, superior viscoelastic properties, and ease of processability. As PCL is a slow-degrading aliphatic polyester which minimizes physiological issues related to local pH shifts due to acidic by-products of metabolism, it has been used in biomaterial and sustainable packaging applications [27–28]. PCL is generally synthesized by ring-opening polymerization (ROP) of cyclic ester ε-caprolactone or polycondensation of 6-hydroxyhexanoic acid. The polycondensation method is not preferred due to the lower quality of products obtained, high degrees of polymerization, and molecular weights above 10 kDa. PCL is soluble at room temperature in numerous solvents including astoluene, benzene, chloroform, cyclohexanone, carbontetrachloride, tetrahydrofuran (THF), dimethyl carbonate (DMC) dioxane, 2-nitropropane, and dichloromethane (DCM) [28].

As understood from the literature review, there have been many studies on obtaining a nanofiber structure by mixing different herbal extracts with polymer materials. The most important points that emerged in these studies was the harmony of fiber morphology between the herbal extract and the polymer material, the interaction between macromolecules, and the formation of a smooth surface. Generally, the percentages of herbal extracts were kept low in the literature (such as 10% w/w in [29], 15%w/v in [23], and 7% w/w in [30]) because extracts could change solution properties. However, if the amount of herbal extract is too low in the nanofiber, they will not be able to demonstrate their antibacterial effects. Therefore, the highest possible amount of herbal extract should be used without disturbing the plant morphology and its antibacterial effects. For this purpose, in this study, mixtures in 3 different amounts (10%, 15%, 25% v/v herbal extract/polymer solution) were prepared and processed through electrospinning. When the produced nanofiber structures were compared, 15% (v/v) of herbal extract/polymer ratio was considered appropriate within the scope of the study. In the study, olive leaves, fumitory, and terebinth plants were used, as they easily grow in Turkey due to its climate. Herbal extracts were obtained by the Soxhlet extraction method and were mixed with PCL and PVA polymers to form a solution. The nanofiber structure was then produced from the prepared solutions using the electrospinning method; characterization, mechanical, and antibacterial tests were performed on these structures. By evaluating these measurements, herbal extract/polymer substance determination with optimum properties was made. Based on the results obtained, nanofiber structures with antibacterial properties that are most appropriate for wound dressing have been proposed.

## 2. Experimental

### 2.1. Material selection

Within the scope of the study, PVA (Mw ∼70000) with product code 843869 was obtained from Merck & Co., Inc. (Kenilworth, NJ, USA), and PCL (Mw ∼45000) with product code 704105 was obtained from Sigma– Aldrich Corp. (St. Louis, MO, USA). In addition, ethanol (Merck), methanol (Merck), chloroform (Merck), acetone (Merck), and distilled water were used.

The plants used were packaged within the last 3 months and were purchased from a local brand, Aktar Diyarı (İzmir, Turkey). The fumitory was obtained from samples collected between May and October. Plants were stored in a cool and dry environment before use.

### 2.2. Preparation of herbal extraction

**Figure 1:**
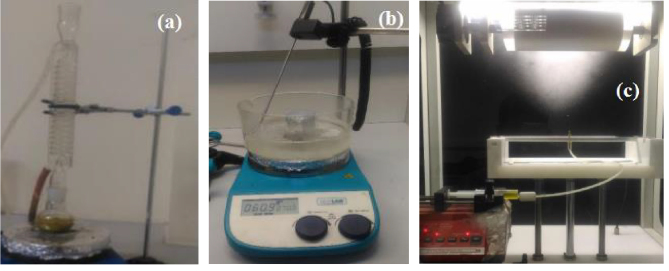
Herbal extraction preparation (a), polymer solution preparation (b), and herbal-based nanofiber production (c).

The plant parts to be used to obtain the chemical components were collected in leaf, root, and/or seed form depending on the plant type. The plant parts were washed twice with distilled water and dried in the drying oven at 70 °C for 10 min in order to remove dust and similar residues. For all 3 plant types, extraction was performed with the Soxhlet extraction method, a method for extracting essential oil from plants. A condenser was used to prevent the loss of solvent with evaporation. The plant extracts were filtered using 2 filter papers and the solvent was removed using an evaporator at 38 °C at 120 rpm rotation (Figure 1a). Herbal extraction preparation parameters are given in Table 1.

**Table 1:** Preparation of herbal extraction.

Plant Type	Extracted Part	Concentration of Extracted Part (%wt)	Solvent Type	Temperature of mixing (° C)	Time of mixing (h)	Stir of mixing (rpm)	References
Olive Leaf	Leaf	25	Methanol/Water (4:1)	Room	24	300	[6]
*Fumaria officinalis*	Haulm and Leaf	10	Methanol	Room	24	300	[20]
*Pistacia terebinthus*	Seed	30	Ethanol	Room	6	300	[31]

### 2.3. Preparation of PVA and PVC polymers

PVA in solid powder form and PCL in bead form were dissolved using a magnetic stirrer for 3 h with the suitable solvent type and ambient conditions indicated in Table 2 (Figure 1b).

**Table 2:** Preparation of pure polymer solutions.

Polymer	Concentration of Polymer (%wt)	Solvent Type (v/v)	Temperature of mixing (°	time of mixing (h)	Stir of mixing (rpm)	References
PVA	14	Distilled water	70	3	500	[32]
PCL	20	Methanol/ Chloroform (1:1)	Room	3	600	[33]

### 2.4. The mixture of polymer/herbal extract solutions

The homogeneous mixing and concentration of the herbal extract/polymer material are two very important factors in activating the antibacterial components in the herbal extract. In this study, mixtures in 3 different proportions (10%, 15%, 25% v/v) were prepared for electrospinning production. Due to the lack of sufficient entanglement or interactions of polymer solutions, discontinuous, uneven, and irregular fiber surfaces were obtained with solutions of 10% (v/v) and 20% (v/v), and it was not possible to produce suitable nanofiber. As a result of the tests conducted, the appropriate concentration of the herbal extract in the polymer has been determined as 15% (v/v), as it gives better results in terms of fiber morphology and chemical properties, especially for antibacterial effects. Eight solutions (A0, AOE, AFO, APT, B0, BOE, BPT, BFO) were prepared for electrospinning with the expansions shown in Table 3 with different matching combinations. These 8 mixture solutions were stirred in the magnetic stirrer for 45 min to ensure homogeneity.

**Table 3:** Production parameters of the electrospinning process.

Coding	Polymer sample	Herbal extract	Feeding rate (mL/h)	Applied voltage (kV)	Distance between syringe needle and collector (cm)	Temperature of production (°C)	Time of production (h)
	A	-	0.5	27	20	24	4
	AOE	*Olea europaea*	0.8	32	20	23	5
A0	AFO	*Fumaria officinalis*	0.9	32	20	22,6	5
	APT	*P. Terebinthus*	0.9	30	20	26,9	5
	B1	-	1.3	29	20	27	3
	BOE	*Olea europaea*	1.2	31	20	26	3
B0	BFO	*Fumaria officinalis*	1.4	29	20	27	3
	BPT	*Pistacia terebinthus*	1.4	28	20	24,6	3

### 2.5. Viscosity and conductivity measurement parameters of polymer/herbal extract solutions

#### 2.5.1. The viscosity measurement of polymer/herbal extract solutions

The viscosities of the 8 polymer solutions were measured using a viscometer (Brookfield DV-E Viscometer, AMETEK Brookfield, Middleboro, MA, USA). The viscosity measurement was made using the S21 spindle type at 100 rpm. The viscosity values of all solutions according to the cutting speed are specified in cP. Two repetitive tests were performed for each measurement.

#### 2.5.2. The conductivity of polymer/herbal extract solutions

Conductivity values of the 8 polymer solutions were measured with a portable electrical conductivity meter (WTW Cond 3110, WTW GmbH, Weilheim, Germany). The conductivity measurement probe was immersed in the prepared mixture solutions and measured in mS/cm.

Viscosity and conductivity measurements were made under laboratory conditions (23 ± 2 °C and 45 ± 10% RH).

### 2.6. Production of herbal extracts loaded nanofibers

After the 8 solutions were prepared and the characterization measurements were taken, 8 mL of solution was taken with a 10-mL syringe. With the syringe placed in the Inovenso NE 300 Nano Spinner electrospinning device (Inovenso Inc., Woburn, MA, USA), nanofiber production was initiated on oil paper by providing the values specified in Table 3 at the rate of 100 rpm (Figure 1c).

### 2.7. Morphology and structure characterizations of herbal extracts loaded nanofibers

Morphology of herbal-based nanofibers was analyzed at 1000× and 20,000× magnification by using SEM (scanning electron microscope) (FEI Sirion XL-30 and Tescan Vega 3; FEI/Thermo Fisher Scientific Inc.„ Waltham, MA, USA and TESCAN USA, Inc., Warrendale, PA, USA). Image J software was used to measure fiber diameters for images, and SPSS 24 statistics software was used to obtain diameter distribution charts. In the FTIR (Fourier transform infrared spectrum) examination of the nanofiber structures obtained, Nicolet is10, a Thermo Scientific brand device, was used. Samples of 10 × 10 mm were taken for both measurements.

### 2.8. Mechanical tests of herbal extracts loaded nanofibers

Measurements of thinness and strength were made to measure the effect of the herbal substance on strength in the plant-based nanofiber structures produced. Thinness measurements were made at 30 different points inboth vertical and horizontal directions with a Mitutoyo Digital Thickness Comparator (Mitutoyo, Kawasaki, Japan). The strength of the samples taken at dimensions of 50 × 10mm (length × width) was measured in an Instron 4411 universal tester (Instron, Norwood, MA, USA).

### 2.9. Antibacterial analysis of herbal extract loaded nanofibers

Antibacterial tests were carried out by using 4 nutrient agars for Gram-negative bacteria *E. coli* (ATCC 35218) and *P. aureginosa* (ATCC 27853) and Gram-positive bacteria *B. subtilis* (ATCC 6633) and S. aureus (ATCC 25293), and by using 1 *C. albicans* medium for *C. albicans* (ATCC 10231) fungi. For bacterial propagation, the sterile medium in McFarland was incubated at 37 °C for 16–24 h after inoculation. After incubation, approximate CFU numbers were calculated with the Biosan Den-1 brand McFarland Densitometer (Biosan Laboratories Inc., Warren, MI, USA). The bacterial culture concentration was produced by incubating at 37 ± 1 °C until the concentration reached 10^8^ CFU/mL.

To nutrient agar medium containing bacteria, 7.5 g nutrient broth, 5 g agar, and 500 mL of water were added, and the mixture was autoclaved. For *C. albicans* , 15 g malt extract, 1.5 g soy peptone, and 7.5 g agar were measured into the autoclave bottle, the volume was increased to 500 mL by adding water, and autoclaving was performed. The media were then divided into sterile Petri dishes with a diameter of 9 cm.

Antibacterial tests were performed with the disc diffusion method according to the ISO 20645: 2004 standard with 2-layer soft agar plates by adding bacteria to the upper layer. Herbal-based nanofiber samples to be tested were cut into 1 × 1cm squares and placed in media containing microorganisms. The bacteriacontaining media were then incubated at 37 °C, and those containing *C. albicans* were incubated at 28 °C for 24 h. The zone diameters formed in Petri dishes were measured using Meazure software.

## 3. Results and discussion

### 3.1. Results of viscosity and conductivity measurement parameters of solutions

Viscosity is the most important parameter in determining the flow rate of a solution. A higher flow rate is provided in electrospinning with low viscosity [34]. In this study, the B0 group samples were found to have lower viscosity values (cP) and higher feeding capacity (mL/h) compared to the A0 group. In addition, the viscosity value is directly related to the molecular weight of the polymer. As the molecular weight of the polymer to be used in the electrospinning process increases, the viscosity of the resulting solution increases [21]. Increasing molecular weight is beneficial for obtaining a smooth morphological surface, but the nozzle may be clogged when working with a compound with very high viscosity. In this study, samples belonging to group A0 showed higher viscosity values than samples of group B0 since they were produced from PVA with higher molecular weight (Table 4).

**Table 4:** The viscosity and conductivity of solutions.

Samples	Conductivity (μS/cm)	Viscosity (cP)	Torque rating (%)
A	854	150	90
AOE	883	208.5	41.7
AFO	665	188	37.6
APT	642	108.5	21.7
B1	0.9	117.5	23.5
BOE	42.8	74	14.8
BFO	4.5	64	12.5
BPT	4.4	71.5	14.3

Another parameter that directly affects the electrospinning process is conductivity. In order to start the electrospinning process, the driving forces in the solution must overcome the surface tension. The water used as a solvent greatly increases conductivity as it allows the molecules to ionize [21]. In this study, it was observed that the samples belonging to the A0 groups provided quite high conductivity properties compared to the B0 group samples without water as the solvent, since water was used as the solvent for the PVA (Table 4).

### 3.2. Determination of morphology and structure characterizations of herbal extract loaded nanofibers

In the study, when the A0 and B0 group nanofiber surface images were compared, it was seen that the surfaces belonging to the A0 group had more surface beading. This is thought to be caused by the lower-viscosity polymer material showing less fiber surface beading on the surface in the electrospinning process [21]. As the viscosity increases, the droplets of solution turn into spindle-like structures and the formation of beaded nanofibers appears [35]. Branched fiber structures were seen in herbal-based nanofiber images for both groups. This is due to the splitting of the solution in forming 2 separate jets at the nozzle, elongation of the jet, changing of the load per unit area carried by jet, and consequent change of the balance between the electrical forces and the surface tension [21]. In the herbal-based nanofiber solutions used in the study, nanofiber surfaces were formed in more branched structures than A and B samples that did not have plant content, since there are 2 different components.

**Figure 2:**
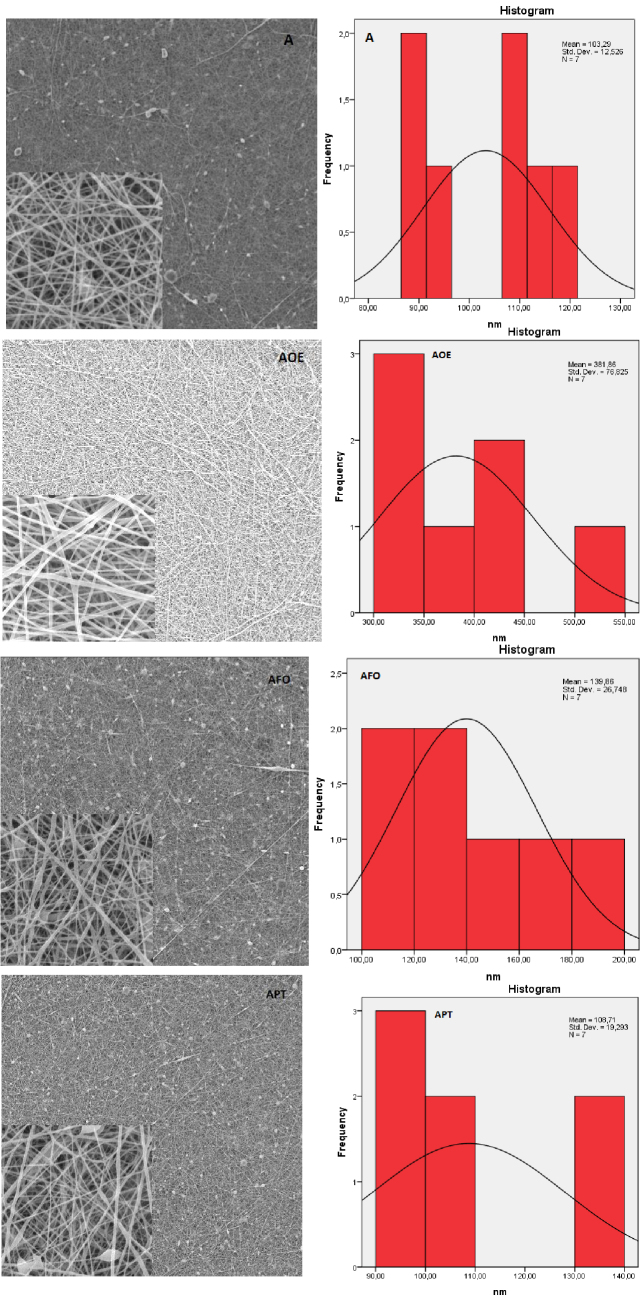
SEM images (1000 and 20,000×) and distribution of diameter (20,000×) of A0 nanofibers.

**Figure 3:**
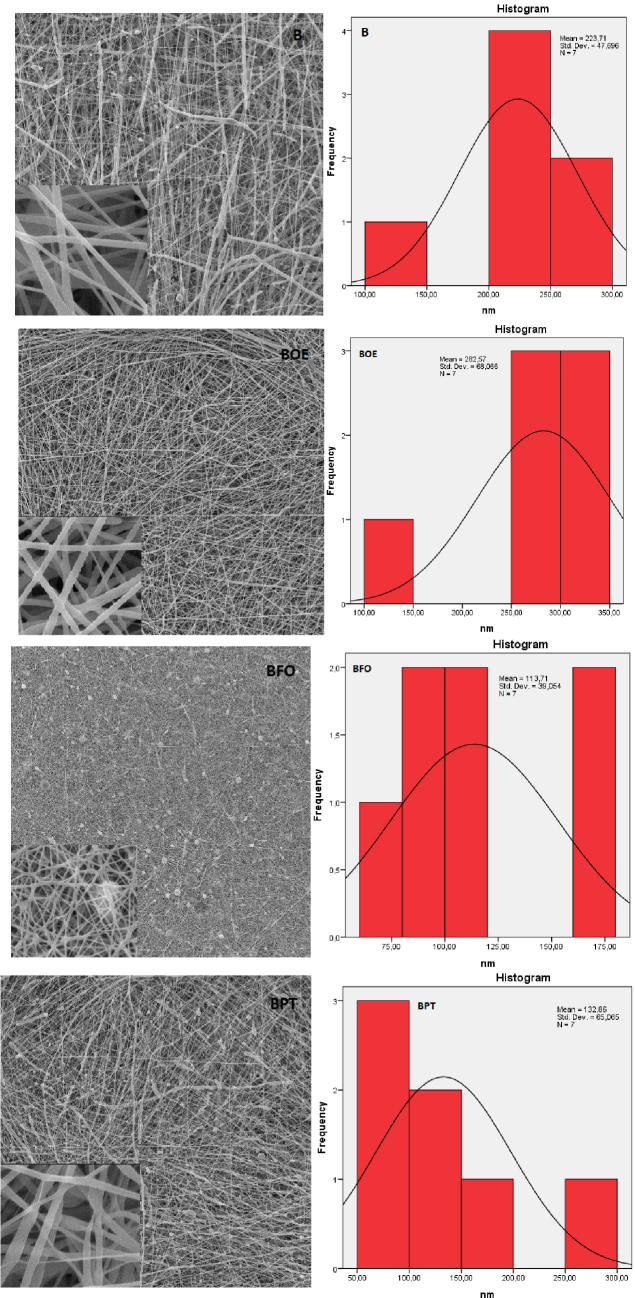
SEM images (1000 and 20,000×) and distribution of diameter (20,000×) of B0 nanofibers.

When the A0 group fiber diameter diagrams were examined in the study (Figure 2), the rank of diameter (nm) measurement in the form of AOE > AFO > APT > A was obtained among the samples. It was observed that the diameter distribution was more homogeneous and uniform in the APT nanofiber structure. For B0 group samples (Figure 3), the BOE > B > BPT > BFO diameter (nm) measurement sequence was observed. As a result of the study, the most uniform structure and the smoothest fiber were obtained in BFO nanofiber. It is thought that APT and BFO nanofiber structures have better morphological features compared to other structures due to the intermolecular interaction between polymer and plant extract.

**Figure 4:**
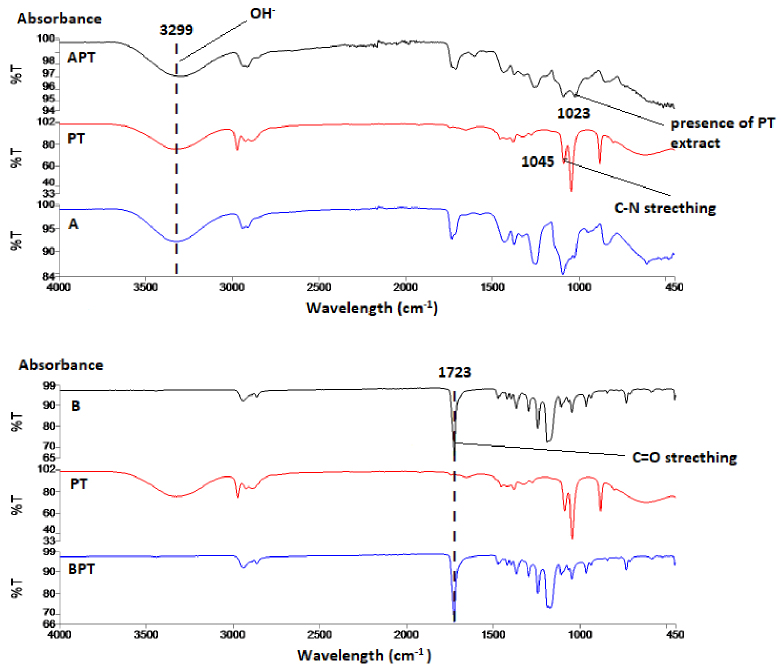
FTIR spectra PT, polymer (A and B), and terebinth/polymer mixture nanofiber structures (APT and BPT).

**Figure 5:**
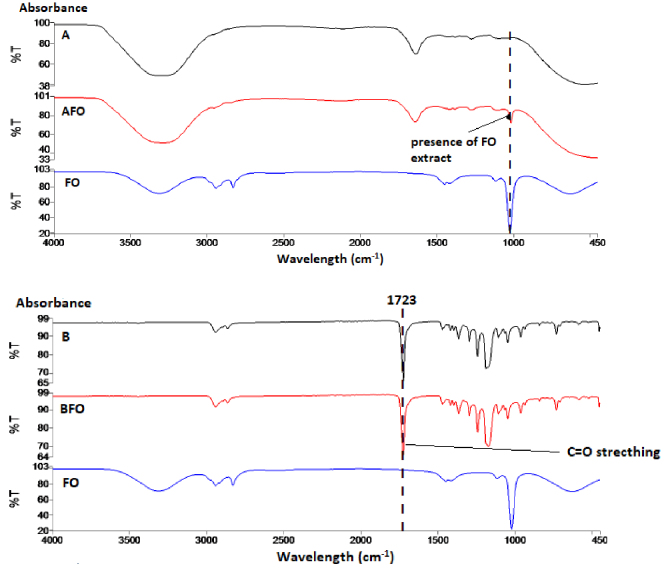
FTIR spectra of the FO, polymer (A and B), and fumitory/polymer mixture nanofiber structures (AFO and BFO).

**Figure 6:**
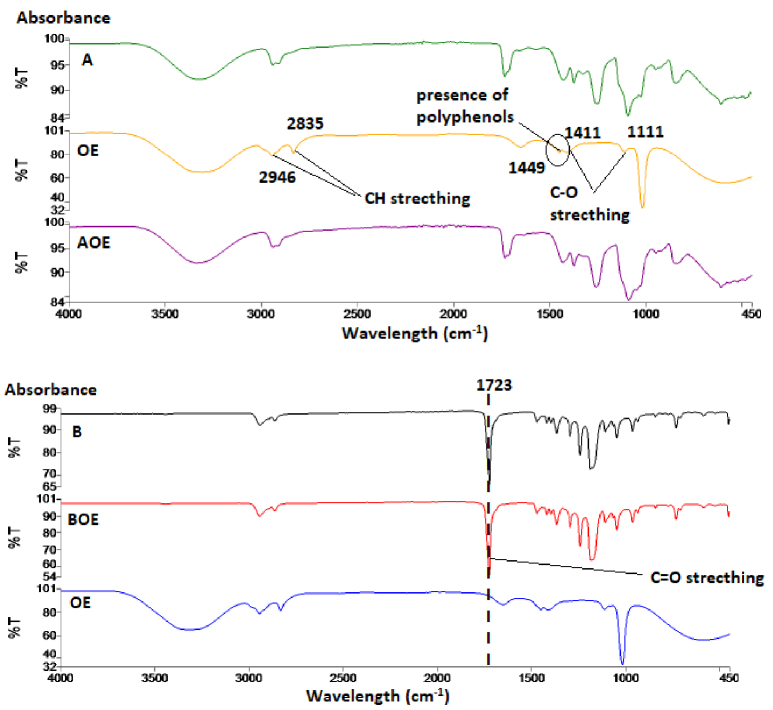
FTIR spectra of OE, polymer (A and B), and olive leaf/polymer mixture nanofiber structures (AOE and BOE).

In all FTIR spectra of the surfaces measured as a result of this study, the wavelength was between 450 and 4000 cm^-1^ for all samples in groups A0 and B0. The band with 3299 cm^-1^ wavelength seen in the PT extract spectrum shows the hydroxyl groups in the nanofiber structure (Figure 4). The hydroxyl group contained in this band is present in polymer A, although it is not present in polymer B. The peak at 1045 cm^-1^ wavelength in PT extract expresses the stretching of C-N bonds [36]. The presence of PT extract at 1023 cm−1 can be seen in the APT nanofiber structure. The absorption band at 1723 cm^-1^ in polymer B expresses C = O stretching [21], and is seen in the BPT nanofiber structure. The reason for the FO extract to show the peak at 1022 cm^-1^ is the presence of =C-H bonds [29]. In the AFO nanofiber structure, the peak of 1015.88 cm^-1^ wavelength indicates the presence of FO extract (Figure 5). Similar to the BPT nanofiber structure, there are C = O stretching at the same absorbance value in the BFO nanofiber spectrum. CH stretches at 2835–2946 cm^-1^ peaks of characteristic bands of OE extract indicates 1111–1411 cm^-1^ absorption zone’s C-O stretching of phenols and the presence of aromatic rings of olive leaf polyphenols at 1449 cm^-1^ [8] (Figure 6). The presence of OE extract at 1015^-1^ cm can be seen in the AOE nanofiber structure. Like the BPT and BFO nanofiber structure, there is C = O stretching at the 1723 cm^-1^ in the BOE spectrum [22].

### 3.3. Strength results of herbal extract loaded nanofibers

**Figure 7:**
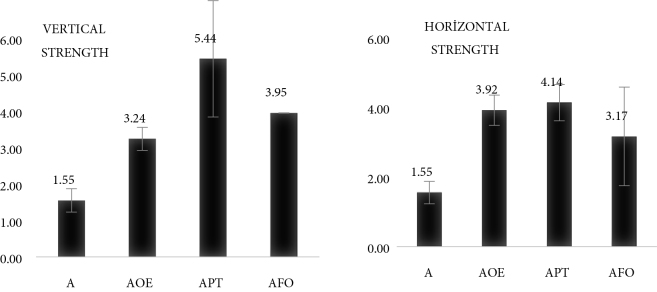
The measured vertical and horizontal strength values (Mpa) of A0 nanofibers.

**Figure 8:**
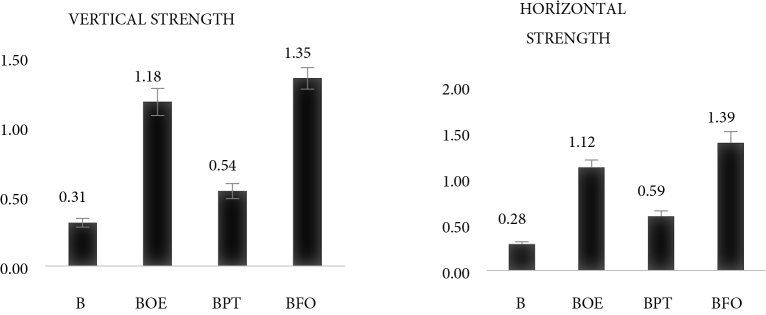
The measured vertical horizontal strength values (Mpa) of B0 nanofibers.

Considering the results of strength measurements applied to nanofiber structures in the study, it was observed that adding herbal extracts to polymer materials increased the strength. In group A0, the highest strength in vertical and horizontal directions was determined on the APT surface (Figure 7). The addition of terebinth extract to the solutions caused a 2.5-fold increase in the strength of the nanofiber structures. In the B0 group samples, the highest strength was observed on the BFO nanofiber surface. The addition of fumitory extract to the solution caused at least a 4-fold increase in the strength of the nanofiber structures (Figure 8). This direct effect of plant extracts on strength is due to their compatibility with the mechanical properties of the polymer texture used [37].

### 3.4. Antibacterial activities of herbal extract loaded nanofibers

**Figure 9:**
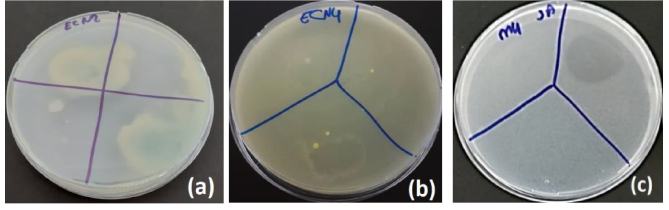
Antibacterial activity effect images of AOE nanofiber against *E. coli* (a), APT nanofiber against *E. coli* (b), and AFO nanofiber against S. aureus (c).

The zone diameters obtained from the antibacterial analysis of herbal-based nanofiber structures by the disc diffusion method are given in Table 5. The determination of antibacterial properties of the produced nanofiber structures was made by comparing them with cefazolin antibiotics, which are widely used in healthcare. In the study, AOE nanofiber showed the highest antibacterial activity against *E. coli* (Figure 9a). According to the results, APT (Figure 9b) and AFO nanofibers were found to be active against *E. coli* . Only AFO nanofiber showed efficacy against *S. aureus* (Figure 9c). AOE, AFO, and APT nanofibers had almost the same inhibition effects on *P. aeruginosa* and *B. subtilis* . On the other hand, the nanofiber sample containing only APT was effective against *C. albicans* . Although there are examples in the literature, an antibacterial effect against *C. albicans* was not obtained with AOE and AFO nanofiber structures. This is thought to be related to some physical and morphological structures, total phenolic components found in plant structures, planting time, plant condition (such as dried or fresh), extracted parts, or the type of solvent [31,32]. Antibacterial release of herbal extracts showed effective properties for all 3 nanofiber structures. Antibacterial effect analysis was not performed for B0 nanofiber structures, assuming that the antibacterial activities of plant extracts would not change.

**Table 5:** The measured zone diameters of nanofibers against bacteria.

	Zones of inhibition (mm)				
Samples	*E. coli*	*P. aeruginosa*	*C. albicans*	*S. aureus*	*B. subtilis*
Cefazolin	52	37		60	
A					
AOE	32	19			18
AFO	17	19		23	21
APT	22	19	28		18

## 4. Conclusion

Within the scope of the study, we aimed to obtain solutions from plant extracts and polymer materials and to measure solution parameters. Nanofibers were produced by electrospinning; morphological structure and spectral analysis, mechanical measurements, and antibacterial tests were performed for the produced nanofibers. In the study, olive leaves, fumitory, and terebinth plants have been used, as they easily grow in Turkey and are utilized in healthcare. As a result of the analysis, it was found that A, APT, AFO, and AOE solutions had higher viscosity and conductivity than B, BPT, BFO, and BOE solutions. This was due to the higher molecular weight of the A0 group samples and the use of water as a solvent. Regarding the morphological analysis, it was observed that APT and BFO nanofibers had the thinnest diameter range and the least beading in the BFO structure. Similarly, APT and BFO nanofiber structures showed the highest strength values in vertical and horizontal strength measurements. The effect of these 2 samples on strength was due to their compatibility with the mechanical properties of the polymer texture used. These results support the results found in the literature [38,21]. When antibacterial properties were examined, it was seen that the 2 samples with the best properties (APT and BFO) were active against 4 different bacteria. However, using BFO against S. aureus and APT against *C. albicans* would increase the efficacy area, since bacterial resistance was high. It is possible to use and develop the nanofiber structures obtained as wound dressing bandages.

In the literature, there were many studies where nanofibers were produced from herbal extracts and used in the fields of drug release, tissue engineering, and wound dressing. However, due to the fact that they are not used extensively, studies on obtaining nanofibers, especially for fumitory and terebinth plants, are limited. Since they are environmentally friendly, biocompatible, sustainable, and economical, these 2 plants have potential to be a source for future biomedical research. It is thought that the use of these plants in the healthcare field for different applications will become widespread. In addition, in future studies, it may be possible to provide new solutions for wound treatments by choosing different plants and suitable polymers with the choice of different rates/variations.
